# Over-Expression of Calpastatin Inhibits Calpain Activation and Attenuates Post-Infarction Myocardial Remodeling

**DOI:** 10.1371/journal.pone.0120178

**Published:** 2015-03-18

**Authors:** Tingqiao Ye, Qiang Wang, Yan Zhang, Xiaofeng Song, Dachun Yang, De Li, Dan Li, Linan Su, Yongjian Yang, Shuangtao Ma

**Affiliations:** Department of Cardiology, Chengdu Military General Hospital, Chengdu, Sichuan, China; University of Western Ontario, CANADA

## Abstract

**Background:**

Calpain is activated following myocardial infarction and ablation of calpastatin (CAST), an endogenous inhibitor of calpains, promotes left ventricular remodeling after myocardial infarction (MI). The present study aimed to investigate the effect of transgenic over-expression of CAST on the post-infarction myocardial remodeling process.

**Method:**

We established transgenic mice (TG) ubiquitously over-expressing human CAST protein and produced MI in TG mice and C57BL/6J wild-type (WT) littermates.

**Results:**

The CAST protein expression was profoundly upregulated in the myocardial tissue of TG mice compared with WT littermates (P < 0.01). Overexpression of CAST significantly reduced the infarct size (P < 0.01) and blunted MI-induced interventricular hypertrophy, global myocardial fibrosis and collagen I and collagen III deposition, hypotension and hemodynamic disturbances at 21 days after MI. Moreover, the MI-induced up-regulation and activation of calpains were obviously attenuated in CAST TG mice. MI-induced down-regulation of CAST was partially reversed in TG mice. Additionally, the MI-caused imbalance of matrix metalloproteinases and their inhibitors was improved in TG mice.

**Conclusions:**

Transgenic over-expression of CAST inhibits calpain activation and attenuates post-infarction myocardial remodeling.

## Introduction

After an acute myocardial infarction (MI), the global heart undergoes a series of structural changes, termed post-infarction myocardial remodeling, leading to the incidence of heart failure [1]. Heart failure secondary to MI remains a major cause of morbidity and mortality. Ventricular remodeling includes the dilatation, hypertrophy, and the formation of a discrete collagen scar. The molecular mechanisms responsible for the post-infarction myocardial remodeling have not been clearly understood [2,3].

The role of proteolytic systems in the development of myocardial remodeling has received much attention [4]. The calpains, a large family of calcium dependent cysteine proteases, have been involved in several kinds of cardiovascular pathophysiological processes [5]. The dysregulation of calpain activity plays an important role in reperfusion injury and myocardial remodeling, suggesting that inhibition of calpain could be a potential therapeutic strategy [6]. The activity of calpain is tightly controlled by calpastatin (CAST), a specific endogenous inhibitor. It has been reported that transgenic over-expression of CAST significantly attenuated angiotensin II-induced myocardial hypertrophy [7], diabetes-related myocardial hypertrophy and fibrosis [8] and endotoxaemia-induced myocardial dysfunction [9]. Over-expression of CAST may also generate a harmful effect, as reported that CAST over-expression enhances doxorubicin-induced cardiac injuries [10]. However, the role of CAST over-expression in post-infarction myocardial remodeling remains poorly understood.

Therefore, the present study established transgenic mice ubiquitously over-expressing CAST and used cultured an *in vivo* model of MI to investigate the role of CAST over-expression in post-infarction myocardial remodeling.

## Materials and Methods

### Ethics Statement

This study was carried out in strict accordance with the recommendations in the Guide for the Care and Use of Laboratory Animals of the National Institutes of Health. The protocol was approved by the Ethics Committee and the Institutional Animal Care and Use Committee of Chengdu Military General Hospital. All surgery was performed under anesthesia, and all efforts were made to minimize suffering.

### Transgenic animal

Transgenic founder mice were generated on a C57BL/6J genetic background. The cDNA from human CAST was cloned into the pDown-CAST vector (Cyagen Biosciences Inc., Guangzhou, China) and verified by sequencing [11]. The linearized pRP.Ex3d-EF1A-CAST-IRES-eGFP was purified from agarose gel using a QIAquick Gel extraction kit (Qiagen, Chatsworth, CA, USA), adjusted to a final concentration of 3 ng/μl in Tris-EDTA buffer and used as a DNA solution for microinjection. The female C57BL/6J mice were hormonally superovulated and mated with male C57BL/6J mice. Next morning the fertilized one-cell eggs were collected from the oviducts. The eggs were microinjected with the DNA solution under a microscope. The injected fertilized eggs were transplanted into the oviducts of pseudo-pregnant C57BL/6J mice. The positive transgenic funder mice were bred with C57BL/6J mice to generate the transgenic mice. Genotyping was performed by polymerase chain reaction with the following primers: CAST: forward 5'-ACG TAA ACG GCC ACA AGT TC-3' and reverse 5'-GAT CTT GAA GTT CAC CTT GAT GC-3', a 440-bp product.

### Plasma glucose and lipids

Fasting plasma glucose (FPG), triglycerides (TG), total cholesterol (TC), low-density lipoprotein-cholesterol (LDL-C) and high-density lipoprotein-cholesterol (HDL-C) of the CAST transgenic (TG) and wild-type (WT) mice were measured using a commercially available kit (Cy-bio Biologic Co., Shangyu, Zhejiang, China) in accordance with the manufacturer's instructions.

### MI protocol

CAST TG and WT mice aged 6–8 weeks were housed under a 12 h/12 h day/night cycle, with *ad libitum* food and water. All surgical procedures were performed using aseptic techniques. MI model was constructed by left anterior descending (LAD) artery ligation as previous described [12]. In brief, mice underwent aseptic lateral thoracotomy after anesthetized with pentobarbital (75 mg/kg by intraperitoneal injection). LAD was permanently ligated with a suture. Sham operated control mice underwent the same surgical procedures except that the suture placed under the LAD was not tied. Twenty-four hours after surgery, surviving mice were monitored and mortality was recorded for 21 days. Twenty-four hours after MI, a part of mice were anesthetized, and myocardial infarct size of hearts was determined by using Evans blue staining (Solarbio, Beijing, China). The rest of mice were euthanized at 4 weeks after MI, and the hearts of mice were harvested and weighed to calculate the heart weight/body weight (HW/BW, mg/g) and left ventricular weight/body weight (LVW/BW, mg/g).

### Hemodynamic analysis

For evaluation of left ventricular hemodynamics, mice were anesthetized by intraperitoneal injection of pentobarbital (75 mg/kg), then a 1.4-F microconductance pressure catheter (ARIA SPR-853; Millar Instruments, Incorporated, Houston, TX, USA) was introduced through the right common carotid artery into the ascending aorta and then advanced into the left ventricle as described previously [13]. Data were collected on Chart via PowerLab (ADInstruments Pty Ltd, Castle Hill, Australia).

### Histological analysis

Hearts were retrograde-perfused with phosphate-buffered saline and were fixed with 10% (v/v) formalin and embedded in paraffin. Paraffin sections (5 μm thickness) were stained with Masson’s trichrome for assessments of infarct scar size and fibrotic area [14]. The positively stained (green) was expressed as a percentage of total area. The left ventricular free wall, interventricular septum and right ventricle were collected as infarct, border and remote zone, respectively. Paraffin sections of interventricular septum were stained with hematoxylin and eosin for assessments of cardiomyocyte hypertrophy.

### Immunohistochemistry

The paraffin heart sections (5 μm thickness) were treated with 3% hydrogen peroxide for 5 min. The sections then were incubated with the primary antibodies: anti-collagen I (1:200 dilution; BA0325, Boster, Wuhan, China) and anti-collagen III (1:200 dilution; BA0326, Boster, Wuhan, China) at 4°C overnight. After being washed, the paraffin sections were incubated with goat anti-rabbit immunoglobulin G biotinylated secondary antibody (1:200 dilution; SA1022, Boster, Wuhan, China) for 1 h at room temperature, and stained using a DAB detection kit (AR1000, Boster, Wuhan, China).

### Western blotting

The proteins of heart were extracted using a protein extraction kit (Keygen Biotech, Nanjing, China). The protein concentrations were determined with an enhanced BCA Protein Assay Kit (Beyotime, Jiangsu, China). Forty micrograms of extracted protein were loaded onto 12% SDS polyacrylamide gels. The separated proteins were then transferred to PVDF membranes. Membranes were blocked with TBS-T containing 5% skim powdered milk for 1 hour and then incubated with anti-calpain-1 (1:200; BA0679, Boster, Wuhan, China), anti-calpain-2 (1:200; BA1575, Boster, Wuhan, China), anti-CAST (1:200; SC-7561, Santa Cruz Biotechnology, Dallas, TX, USA), anti-matrix metalloproteinase (MMP)-2 (1:200; BA0569, Boster, Wuhan, China), anti-MMP-9 (1:200; BA2202, Boster, Wuhan, China), anti-tissue inhibitor of MMP (TIMP)-1 (1:200; BA3727, Boster, Wuhan, China), anti-TIMP-2 (1:200; BA0576, Boster, Wuhan, China), anti-TGF-β (1: 500; ab66043, Cambridge, MA, USA) and GAPDH (1:200; BA2913, Boster, Wuhan, China) antibodies overnight. Membranes were rinsed three times with TBS-T and incubated with horseradish peroxidase-conjugated goat anti-rabbit immunoglobulin G (1:1000; A0208, Beyotime, Jiangsu, China) for 1 hour. Membranes were rinsed three times with TBS-T. Chemiluminescence detection reagent (BeyoECL Plus, P0018, Beyotime, Jiangsu, China) were dropwise added on the membranes. The luminescent signal was detected by exposure to x-ray film.

### Calpain activity

Calpain activity was determined by using a fluorescence substrate N-Succinyl-Leu-Leu-Val-Tyr-7-amido-4-methylcoumarin (Suc-LLVY-AMC) with a calpain activity assay kit (Genmed scientifics inc., Shanghai, China) as described in a previous study [15].

### Real-time polymerase chain reaction (RT-PCR)

The mRNA expression of mouse endogenous CAST in the heart tissue of WT and TG mice was detected by RT-PCR. The RT-PCR was performed using One Step SYBR Prime Scrip RT-PCR Kit II (RR086A, TaKaRa). The relative amount of mRNA was calculated by 2^−ΔΔCT^ and was normalized to a housekeeping gene 18s rRNA. Each sample was run and analyzed in triplicate. The average of the relative amount of each mRNA in WT group was defined as 1.0. PCR primer sequence is listed as follow: CAST: F, 5’-GAG CAG TCA GCC TTC CAG AC-3’; R, 5’-TCT GTG GTA CTC ATG CTG GG-3’; 18s rRNA: F, 5’-CGC GGT TCT ATT TTG TTG GTT T-3’; R: 5’-GCG CCG GTC CAA GAA TTT-3’.

### Statistical analysis

Continuous data are presented as mean ± standard error (SE). Comparisons between the groups were determined by ANOVA with *post-hoc* t-tests (SPSS Inc., Chicago, IL, USA). Survival curves were created by the method of Kaplan and Meier, and compared by log-rank test. P < 0.05 was considered statistically significant.

## Results

### Establishment of transgenic mice

**Fig 1:**
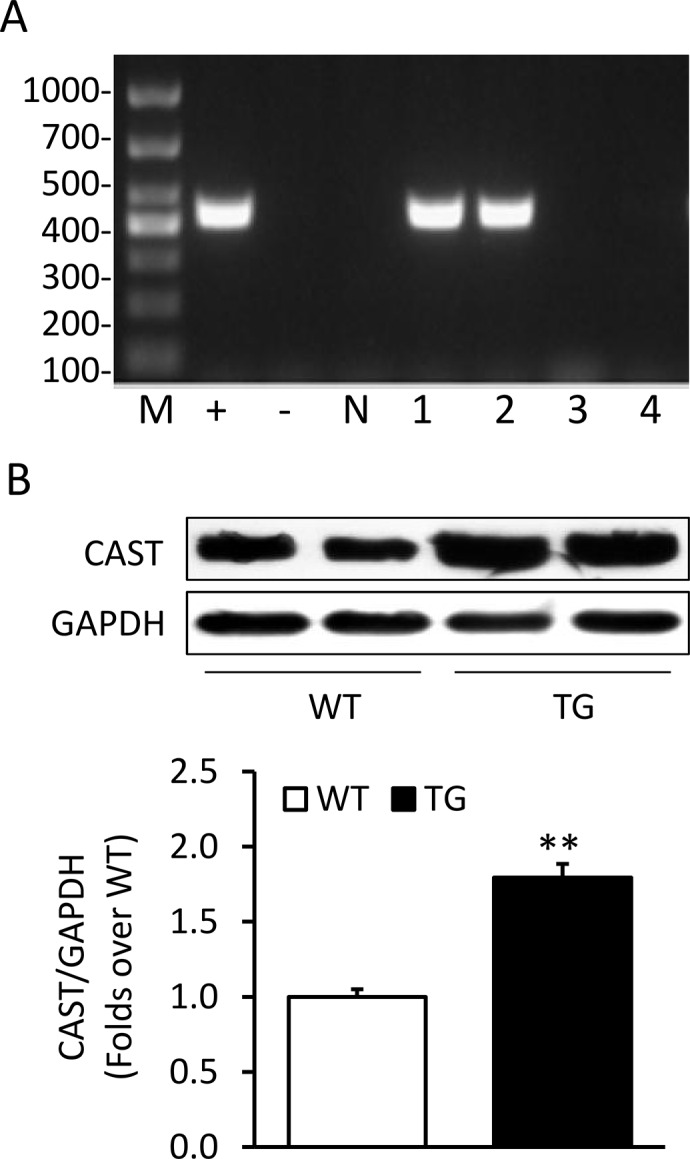
Establishment of transgenic mice. (A) Genotyping using PCR method of transgenic mice. M, marker; +, positive control;-, blank; N, negative control; 1 and 2, positive transgenic mice; 3 and 4, negative mice. (B) Western blotting of calpastatin (CAST) in myocardial tissue from transgenic (TG) mice and wild-type (WT) littermates. Data are expressed as means ± SE. n = 6. **P<0.01 vs. WT group.

**Fig 2:**
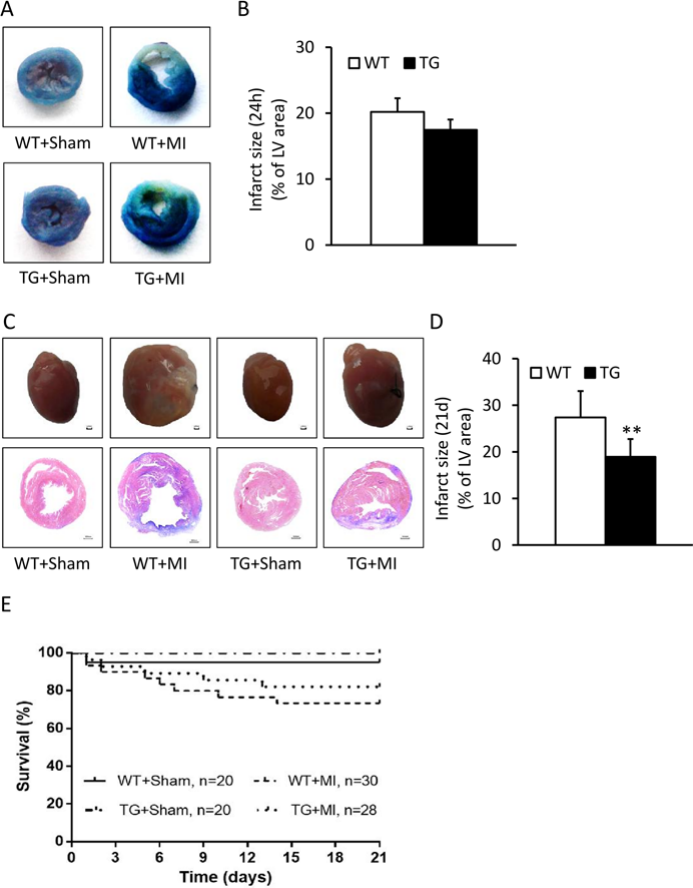
Over-expression of CAST reduces infarction size. (A) Representative Evans blue stained hearts from the calpastatin (CAST) transgenic (TG) mice and wild-type (WT) littermates at 24 hours after surgery. (B) Quantification of infarct size at 24 hours after surgery. (C) Gross hearts and hematoxylin & eosin staining of CAST TG mice and WT littermates in sham and myocardial infarction (MI) group. (D) The infarct size of left ventricle for the TG mice and WT mice. (E) Survival of the mice. Data are expressed as means ± SE. **P < 0.01 vs. WT group.

The transgenic fragments containing the human CAST cDNA were microinjected into the male pronuclei of 150 fertilized oocytes of C57BL/6J mice. A total of 90 injected eggs were implanted into the oviducts of 3 pseudo-pregnant foster mothers, which gave birth to 23 offspring. Two offspring mice were identified to be carrying the CAST cDNA by PCR analysis (Fig. 1 A). Western blotting analysis confirmed that the CAST protein was significantly over-expressed in the heart of the TG mice compared with the WT mice (Fig. 2B). The transgenic over-expression did not affect the mRNA expression of endogenous CAST (S1 Fig). The FPG, TG, TC, LDL-C and HDL-C were similar between the TG mice and WT littermates (Table 1).

**Table 1:** Characteristics of the transgenic mice.

	WT	TG
BW (g)	23.11 ± 0.69	23.17 ± 1.13
FPG (mmol/L)	5.42 ± 0.24	5.22 ± 0.33
TG (mmol/L)	0.40 ± 0.02	0.43 ± 0.02
TC (mmol/L)	5.00 ± 0.29	5.41 ± 0.24
LDL-C (mmol/L)	4.45 ± 0.25	5.14 ±0.26
HDL-C (mmol/L)	0.57 ± 0.03	0.54 ± 0.03

Values are means + SE. n = 6. WT, wild-type mice; TG, Calpastatin transgenic mice; BW, body weight; FPG, fasting plasma glucose; TG, triglyceride; TC, total cholesterol; LDL-C, low density lipoprotein-cholesterol; HDL-C, high density lipoprotein-cholesterol.

### Over-expression of CAST reduces infarction size

The infarct size at 24 hours after surgery has no difference between the TG and WT mice (Fig. 2 A-B). No infarction was detected in sham hearts. Compared with hearts from WT mice, transgenic overexpression of CAST limited infarct size (18.9 ± 3.8% vs. 27.4 ± 5.6%, P < 0.01, Fig. 2 C-D). The survival rate has no differences between the TG mice and WT littermates (Fig. 2 E).

### Over-expression of CAST preserves cardiac hemodynamics

MI produced a hypotension status in WT mice, which was partially reserved by transgenic expressing CAST (Table 2). Hemodynamic analysis revealed that MI significantly decreased the left ventricular end-diastolic pressure, increased the left ventricular end-systolic pressure, and decreased maximal rate of pressure development and maximal rate of pressure decay in WT mice (all P<0.01, Table 2). However, these effects were significantly attenuated in CAST TG mice (P<0.01 or P < 0.05, Table 2).

**Table 2:** Hemodynamic parameters of mice.

	WT		TG	
	Sham (*n* = 7)	MI (*n* = 8)	Sham (*n* = 6)	MI (*n* = 7)
SBP (mmHg)	102.8 ± 5.09	69.55 ± 3.27**	103.30 ± 4.39	82.84 ± 4.99^#^
DBP (mmHg)	82.26 ± 4.51	45.99 ± 2.44**	86.76 ± 4.82	63.00 ±3.39^##^
MAP (mmHg)	89.47 ± 4.62	56.18 ± 3.72**	92.49 ± 4.49	69.33 ± 4.38^#^
LVESP (mmHg)	72.43 ± 2.99	46.60 ± 2.60**	74.81 ± 3.81	57.88 ± 2.76^#^
LVEDP (mmHg)	2.65 ± 0.93	18.80 ± 1.07**	1.67 ± 0.91	10.48 ± 0.96^##^
dP/dt_max_ (mmHg/s)	2684 ± 183	693 ± 106**	2798 ± 200	1803 ± 185^##^
dP/dt_min_ (mmHg/s)	-2041 ± 184	-378 ± 75**	-2061 ± 94^#^	-1217 ± 96^##^

Values are means + SEM. WT, wild-type mice; TG, Calpastatin transgenic mice; MI, myocardial infarction; SBP, systolic blood pressure; DBP, diastolic blood pressure; MAP, mean arterial pressure; LVESP, left ventricular end-systolic pressure; LVEDP, left ventricular end-diastolic pressure; dP/dt_max_, maximal rate of pressure development; dP/dt_min_, maximal rate of pressure decay.

**P < 0.01 *vs*. WT+Sham;

^#^P < 0.05,

^##^P < 0.01 *vs*. WT+ MI.

### Over-expression of CAST attenuates hypertrophy and fibrosis

**Fig 3:**
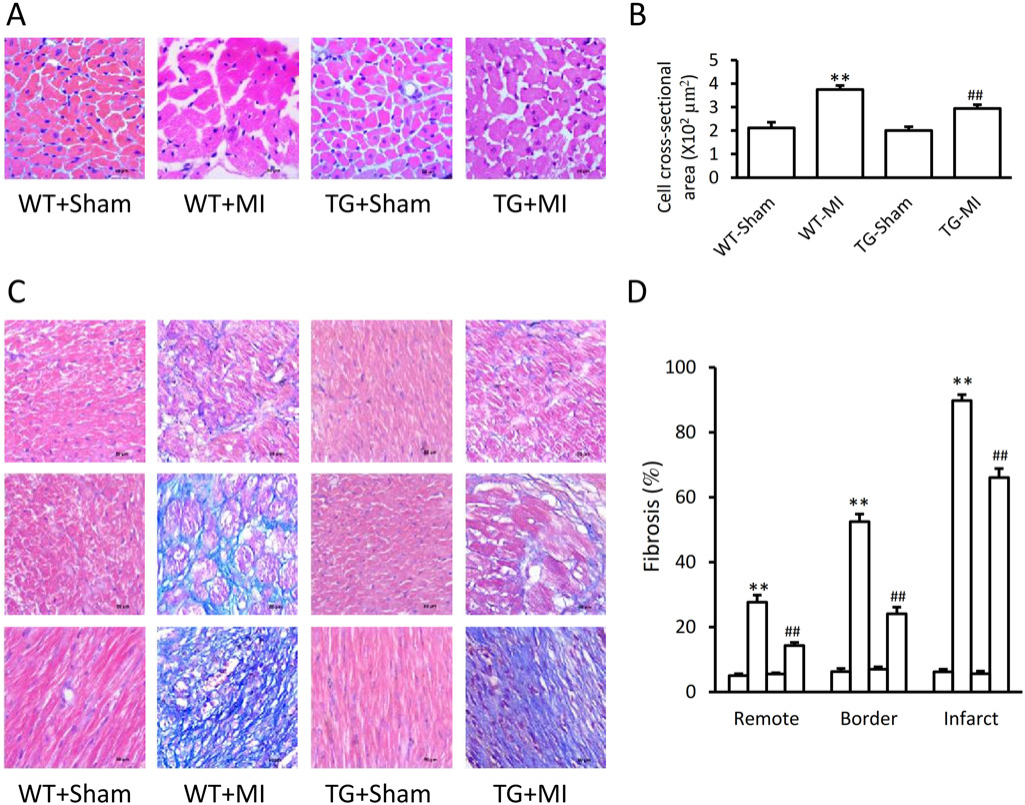
Over-expression of CAST attenuates hypertrophy and fibrosis. (A) Representative hematoxylin & eosin stained cross-sections of interventricular septum from the calpastatin (CAST) transgenic (TG) mice and wild-type (WT) littermates subjected to sham surgery and left anterior artery occlusion. (B) Quantification of cardiomyocyte cross-sectional area. (C) Representative Masson stained heart sections of remote (upper panel), border (middle panel), and infarct (lower panel) zone from the four groups of the mice. (D) Quantification of fibrosis. Data are expressed as means ± SE. **P<0.01 vs. WT-Sham group, ^##^P<0.01 vs. WT-MI group.

**Fig 4:**
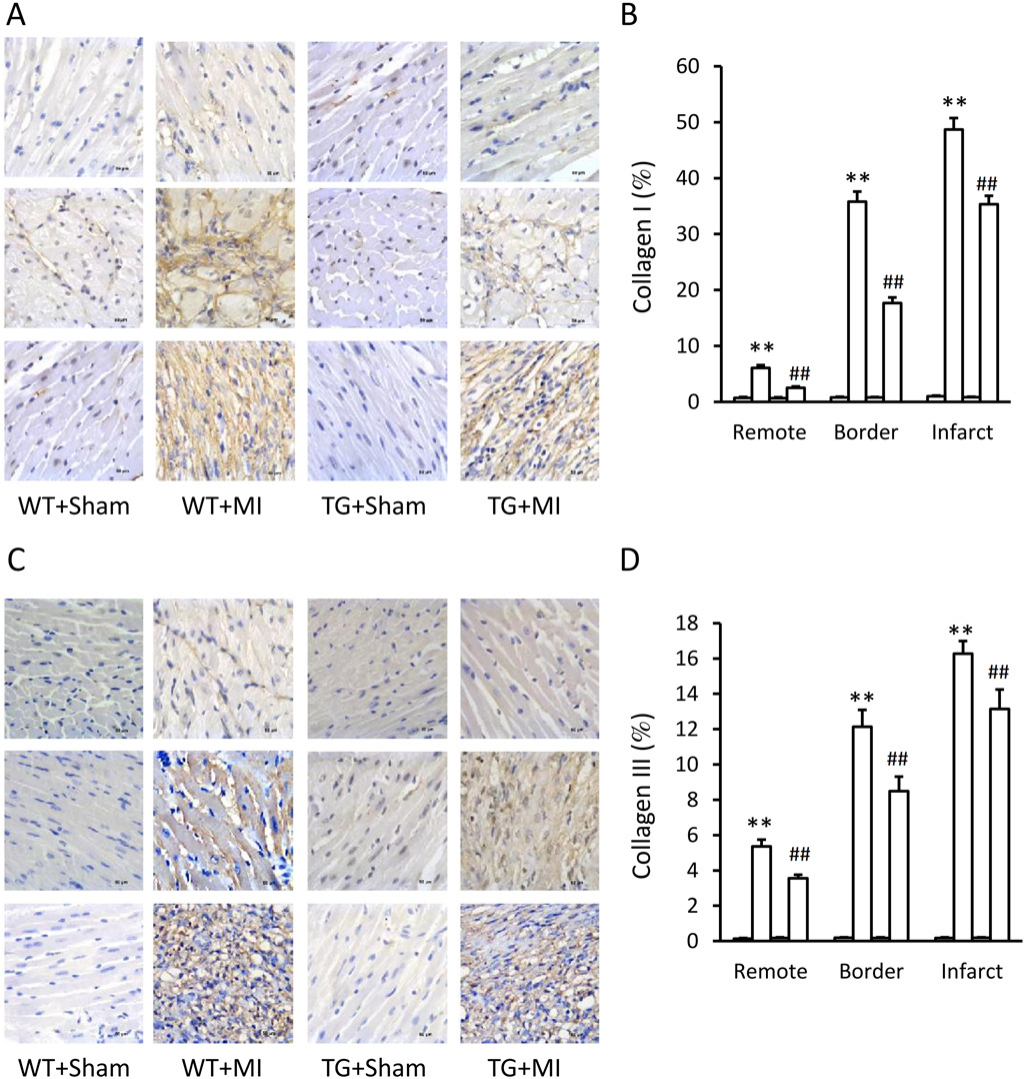
Over-expression of CAST decreases collagen deposition. Representative immunohistochemical images of collagen I (A) and collagen III (C) in heart sections of remote (upper panel), border (middle panel), and infarct (lower panel) zone from the calpastatin (CAST) transgenic (TG) mice and wild-type (WT) littermates subjected to sham and MI surgery. Quantification of positive collagen I (B) and collagen III (D) staining. Data are expressed as means ± SE. **P<0.01 vs. WT-Sham group, ^##^P<0.01 vs. WT-MI group.

The HW/BW ratio was significantly increased in MI hearts from WT mice (4.28 ± 0.18 *vs*. 6.56 ± 0.16 mg/g, P<0.01) and TG mice (4.51 ± 0.2 *vs*. 5.34 ± 0.2 mg/g, P<0.01). However, the increase in HW/BW ratio was blunted in TG mice (P<0.01). Histological analysis revealed that MI caused significant cardiomyocytic hypertrophy of interventricular septum and remarkable cardiac fibrosis of infarct area, border zone, and even remote region (Fig. 3 A-B). However, these pro-hypertrophic and pro-fibrotic effects were obviously blunted by over-expressing of CAST in TG mice (Fig. 3 A-B). Similar results were obtained in the immunohistochemical assays of collagen I and collagen III in the myocardium of infarct area, border zone, and remote region (Fig. 4 A-B).

### Over-expression of CAST decreases calpain expression and activity

**Fig 5:**
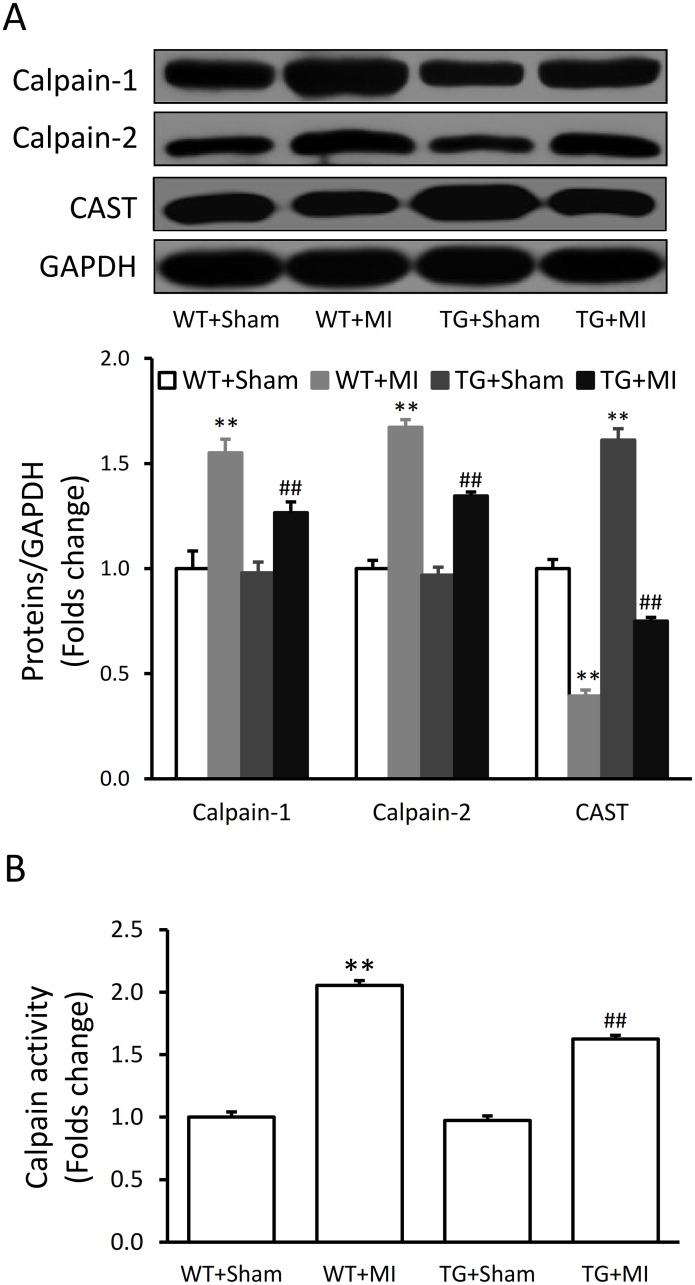
Over-expression of CAST suppresses calpain expression and activity. (A) Representative Western blots of calpain-1, calpain-2 and calpastatin (CAST) of the heart tissue from the CAST transgenic (TG) mice and wild-type (WT) littermates subjected to sham and myocardial infarction (MI) surgery. (B) Calpain activity in the myocardial tissue. Data are expressed as means ± SE. **P<0.01 vs. WT-Sham group, ^##^P<0.01 vs. WT-MI group.

The protein expressions of calpain-1 and calpain-2 in myocardial tissue were significantly increased and the expression of CAST was significantly decreased after MI in WT mice, and these effects were attenuated in TG mice (Fig. 5 A). Similarly, the activity of calpain was increased after MI in WT mice (Fig. 5 B). Transgenic over-expressing of CAST did not change the baseline but significantly attenuated MI-induced activation of calpains (Fig. 5 B).

### Over-expression of CAST regulates MMP/TIMP system

**Fig 6:**
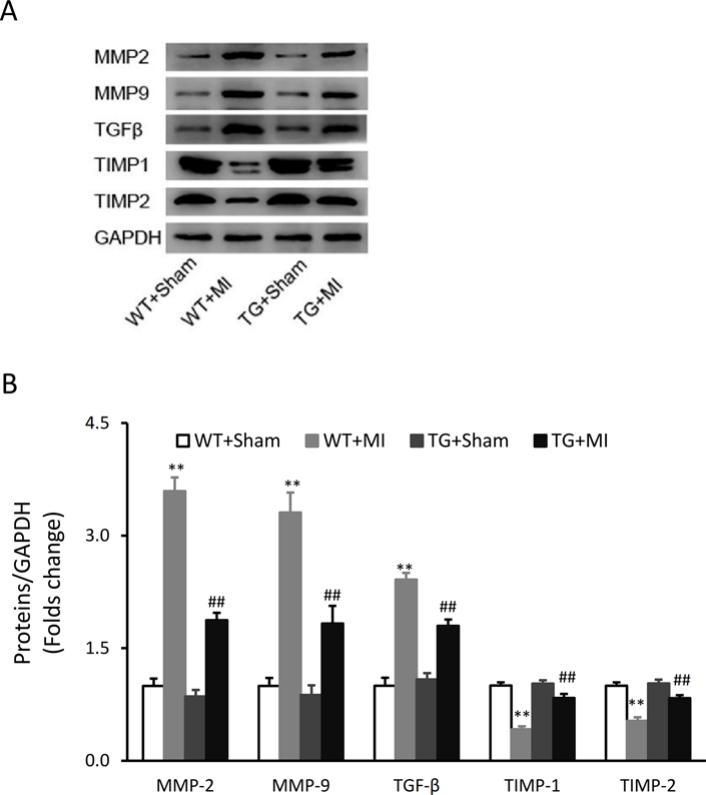
Over-expression of CAST regulates MMP/TIMP system. (A) Representative Western blots of matrix metalloproteinase (MMP)-2, MMP-9, transforming growth factor-β (TGF-β), tissue inhibitor of MMP (TIMP)-1 and TIMP-2 of the heart tissue from the CAST transgenic (TG) mice and wild-type (WT) littermates subjected to sham and MI surgery. (B) Bar graph shows the quantification of Western blots. Data are expressed as means ± SE. **P < 0.01 vs. WT-Sham group, ^##^P < 0.01 vs. WT-MI group.

MI induced significant increases in the protein expression of MMP-2, MMP-9, TGF-β and significant decreases in TIMP-1 and TIMP-2 in WT mice (Fig. 6 A-B). However, these effects were significantly attenuated in CAST TG mice (Fig. 6 A-B).

## Discussion

In the present study, we established a transgenic mouse model overexpressing human CAST and provided the experimental evidence that post-infarction myocardial remodeling is associated with up-regulation and activation of calpains and down-regulation of CAST. Transgenic over-expression of CAST significantly attenuated MI-induced calpains upregulation and activation and blunted post-infarction myocardial remodeling.

The pathophysiological role of calpains and CAST system in MI hearts has remained unclear despite several excellent studies. Since previous studies had shown that the expressions and activities of calpains were increased in the hearts following MI [16–18], inhibiting of calpain system became a potential strategy for the treatment of post-infarction ventricular remodeling. Genetic ablation of calpain regulatory subunit [19] or pharmacologically inhibition of calpain significantly [20] reduces adverse myocardial remodeling and myocardial dysfunction after MI. However, the role of CAST, the intrinsic inhibitor of calpains, in infarcted hearts received less attention. A recent study demonstrated that calpain-mediated proteolysis was enhanced in the heart following MI and that profound activation of calpains exacerbated ventricular remodeling in CAST-deficient mice [21]. However, the ablation of CAST didn’t affect the prevalence of cardiomyocyte apoptosis, hinting that CAST may affect the matrix remodeling by inhibiting the photolytic activity of calpains [21]. From another perspective, the present study confirms the protective role of CAST in the infarcted hearts by using a gain-of-function strategy. Our study suggests that transgenic over-expression of CAST attenuates MI-induced increases in the expression and activity of calpains and subsequently suppresses calpains-caused cardiac matrix remodeling, including chamber dilatation, fibrosis and cardiac dysfunction. The infarct size was indistinguishable between CAST^-/-^ and CAST^+/+^ mice in the previous report [21]. We found the infarct size at 24 hours after surgery was similar between CAST TG mice and WT littermates. However, we found the infarct size was decreased in CAST TG mice compared with WT littermates at 21 days after MI. This phenomenon may be attributed to the extension of the infarct area due to the degradation of extracellular matrix.

Although the beneficial effect against post-infarction remodeling has been reported in mice lacking capn4 that may impair the activity of calpain-1 and calpain-2 [19], the present study focuses on the beneficial role of CAST. The role of CAST may be mediated by impairing calpains and may also be mediated by indirectly inhibiting MMP activity. The present study also suggests that overexpression of CAST via vector delivery system may be a therapeutic strategy for post-infarction remodeling.

MI not only caused a profound fibrosis of infarct zone, but also induces a significant fibrosis in border and remote zone of the heart, which might be due to the activation of local neurohormones such as renin-angiotensin system in the heart. It is well established that local renin-angiotensin system is activated during post-infarction ventricular remodeling process and the angiotensin II is an important upstream activator of calpains [7]. Thus, the global activation of calpains induced by angiotensin II might contribute to the fibrosis far from the infarct zone.

MMPs and TIMPs system is an important regulator of matrix remodeling. It has been reported that MMP/TIMP system can be regulated by calpains [8]. The present study demonstrated that overexpression of CAST can ameliorate MI-induced imbalance of MMP/TIMP system, indicating that the normalization of MMP/TIMP system could be a potent mechanism underlying the action of CAST. However, the mechanisms for the beneficial role of CAST have not yet been fully investigated. The further studies focused on seeking the targets of CAST may help us understanding the mechanisms.

Collectively, the present study indicates that over-expression of CAST attenuates MI-induced activation of calpains and subsequently ameliorates post-infarction myocardial remodeling.

## Supporting Information

S1 Fig The mouse endogenous CAST mRNA expression. The mRNA expression of mouse endogenous CAST in heart tissue from CAST TG and WT mice. Data are expressed as means ± SE. n = 3 each group.
(TIF)
