# Supplementary figures and images for: Over-Expression of Calpastatin Inhibits Calpain Activation and Attenuates Post-Infarction Myocardial Remodeling
Source: PLoS One. 2015 Mar 18;10(3):e0120178. doi: 10.1371/journal.pone.0120178 (PMC4364764; doi:10.1371/journal.pone.0120178)

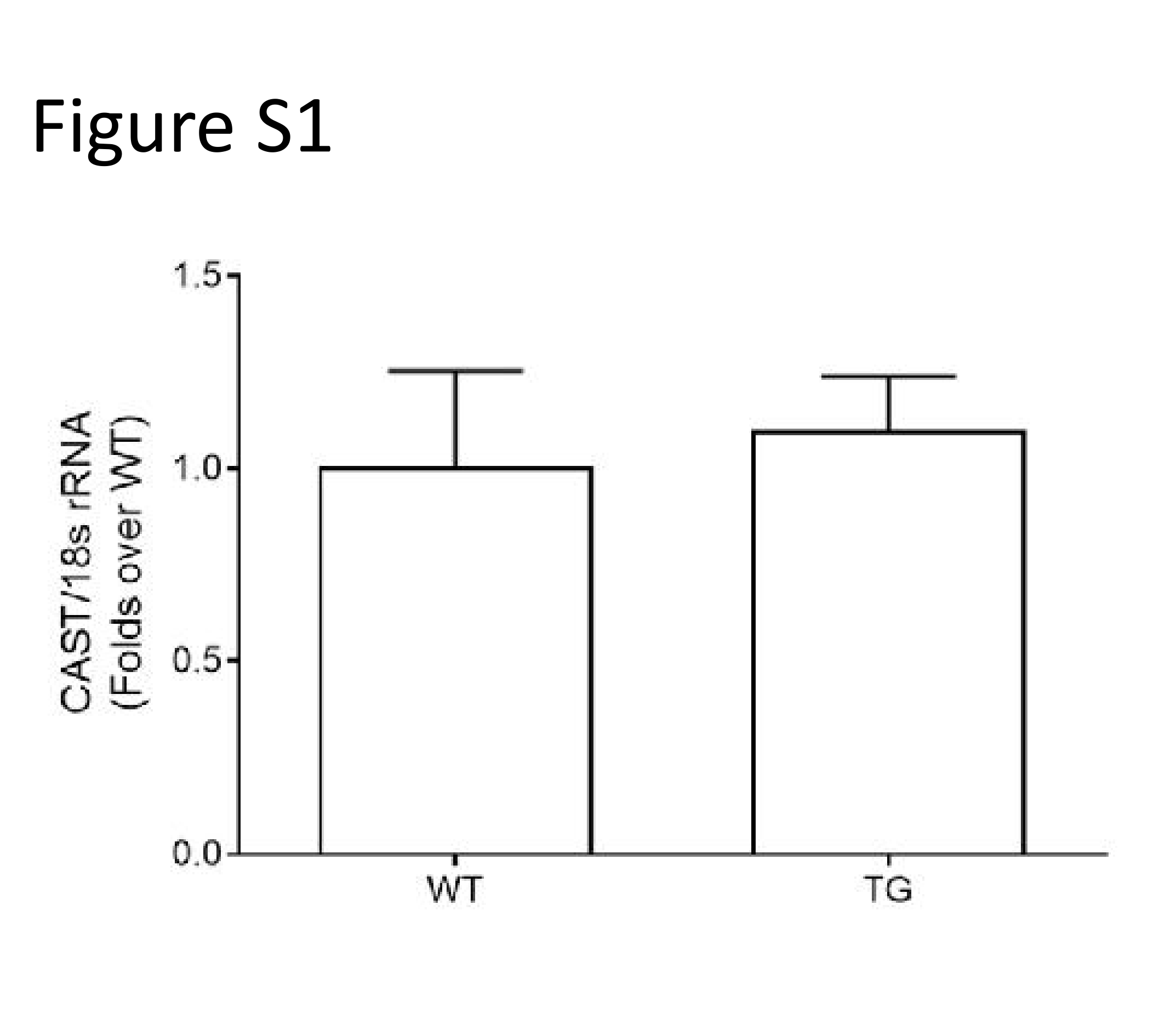

Supplement: S1 Fig — The mRNA expression of mouse endogenous CAST in heart tissue from CAST TG and WT mice. Data are expressed as means ± SE. n = 3 each group. (TIF) [file pone.0120178.s001.tif]
